# Characterization and Fine Mapping of *ds*, a Recessive Dense-Spike Mutant Associated with Shortened Spike Axis and Increased Spikelet Number in Wheat

**DOI:** 10.3390/plants15162470

**Published:** 2026-08-14

**Authors:** Xiangtai Che, Zhuo Li, Shaoyuan Chen, Luxue Liu, Jie Ning, Jinwei Feng, Xin Wang, Yanhu Guo, Haotong Sun, Qingquan Chen, Jiancheng Song, Jing Zhao, Lei Chen

**Affiliations:** School of Life Sciences, Yantai University, Yantai 264005, China; chexiangtai2024@163.com (X.C.); lz000826@163.com (Z.L.); q13114858850@163.com (S.C.); x11498372@163.com (L.L.); njlyf1014@163.com (J.N.); 17865170952@163.com (J.F.); 15653427627@163.com (X.W.); 15725352129@163.com (Y.G.); 15645832002@163.com (H.S.); 18263568195@163.com (Q.C.); jcsong88@163.com (J.S.)

**Keywords:** wheat, EMS mutagenesis, dense-spike mutant, spike architecture, molecular marker, fine mapping, gibberellin, transcriptome

## Abstract

Spike density is an important component of wheat spike architecture and is determined by the combined effects of spike-axis elongation and spikelet number. In this study, we characterized an ethyl methanesulfonate (EMS)-induced recessive dense-spike mutant, *ds*, which was obtained from EMS mutagenesis of the wheat cultivar YN21 in 2013 and displays a field-visible compact spike architecture associated with a shortened spike axis, reduced spike internode spacing, and increased spikelet number. Genetic analysis showed that all F_1_ plants exhibited normal spikes and that segregation in the F_2_ population fitted a 3:1 ratio, supporting control by a single recessive nuclear gene. Through genome-wide marker-based linkage screening, enlarged-population validation, and fine mapping, *ds* was delimited to an approximately 864.819 kb interval between ID2B2796 and ID2B7615 on chromosome 2B. This interval contains 11 high-confidence annotated genes, including F-box protein, actin, ubiquitin-conjugating enzyme, ribosomal protein L16, RecX, plant cysteine oxidase, PI4KIIγ, and two adjacent *GA3OX*-family-related genes. Integrated RNA-seq and RT-qPCR analyses showed that the two adjacent *GA3OX*-family-related genes were expressed in young spikes and exhibited reduced expression in *ds*-sib relative to WT-sib. These expression data support their retention as plausible, non-exclusive candidates but do not establish causality. Transcriptome analysis further revealed changes in hormone-related pathways, cell-wall organization, carbohydrate metabolism, and transcription-factor regulation. These results provide a reliable genetic basis for further molecular cloning of *ds* and suggest that altered hormone- and growth-related transcriptional responses may be associated with dense-spike formation in wheat.

## 1. Introduction

Spike architecture is a critical determinant of grain yield in wheat (*Triticum aestivum* L.), because it influences both grain number and the spatial arrangement of spikelets within the inflorescence [1,2,3,4,5]. Among the major spike-related traits, spike density, defined as the number of spikelets per unit spike length, is especially important because it integrates changes in rachis elongation, spikelet arrangement, and overall plant architecture [6,7,8,9,10].

Genetic studies have identified several key regulators of wheat spike morphology. The domestication gene *Q*, which encodes an APETALA2-like transcription factor, plays a central role in spike compactness, rachis fragility, and threshability [6]. Recent work has shown that genes affecting spikelet number, basal spikelet development, and inflorescence determinacy also contribute substantially to spike architecture and yield potential, including loci associated with *TB1*, *FT-B1*, *VRT-A2*, *WAPO-A1*, *SPL* genes, and *FZP*-related pathways [3,4,8,9,10,11,12,13]. These studies indicate that dense or compact spike phenotypes may arise from altered spikelet initiation, modified meristem determinacy, or reduced longitudinal growth of the spike axis.

Phytohormones are central regulators of inflorescence growth and plant architecture. Gibberellins (GAs) are major promoters of stem and internode elongation, and defects in GA biosynthesis or signaling frequently lead to dwarf or compact phenotypes [14,15,16,17]. Auxin controls growth polarity, organ patterning, and cell expansion, often through interactions with transporters and downstream transcriptional regulators [18,19]. Hormone crosstalk, including interactions among GA, auxin, and brassinosteroid pathways, is widely implicated in shaping plant architecture and internode elongation [20,21].

Cell-wall remodeling and carbohydrate metabolism are also major determinants of organ elongation. Cell expansion requires coordinated modification of the primary cell wall, including changes in cellulose–hemicellulose interactions, pectin status, wall-loosening activity, and wall-integrity signaling [22,23,24,25,26]. Because spike density is structurally associated with rachis internode length, alterations in cell-wall dynamics provide a plausible mechanistic link between hormonal regulation and compact spike morphology.

In the present study, we characterized an EMS-induced recessive dense-spike mutant, *ds*, obtained from an EMS-mutagenized population of the wheat cultivar YN21 generated in 2013. The *ds* phenotype is visually distinguishable under field conditions and is characterized by a shortened spike axis, increased spikelet number, and reduced spike internode spacing. We combined genome-wide marker-based linkage screening, enlarged-population validation, fine mapping, RNA-seq, RT-qPCR, and tissue-expression profiling to delimit *ds* to an 864.819 kb interval on chromosome 2B. The genes located in this interval were considered collectively, and candidate prioritization was performed cautiously based on position, functional annotation, and expression evidence. This study provides a genetic foundation for further cloning of *ds* and offers insight into the regulation of wheat spike compactness.

## 2. Results

### 2.1. Phenotypic Characterization of the ds Mutant

**Figure 1:**
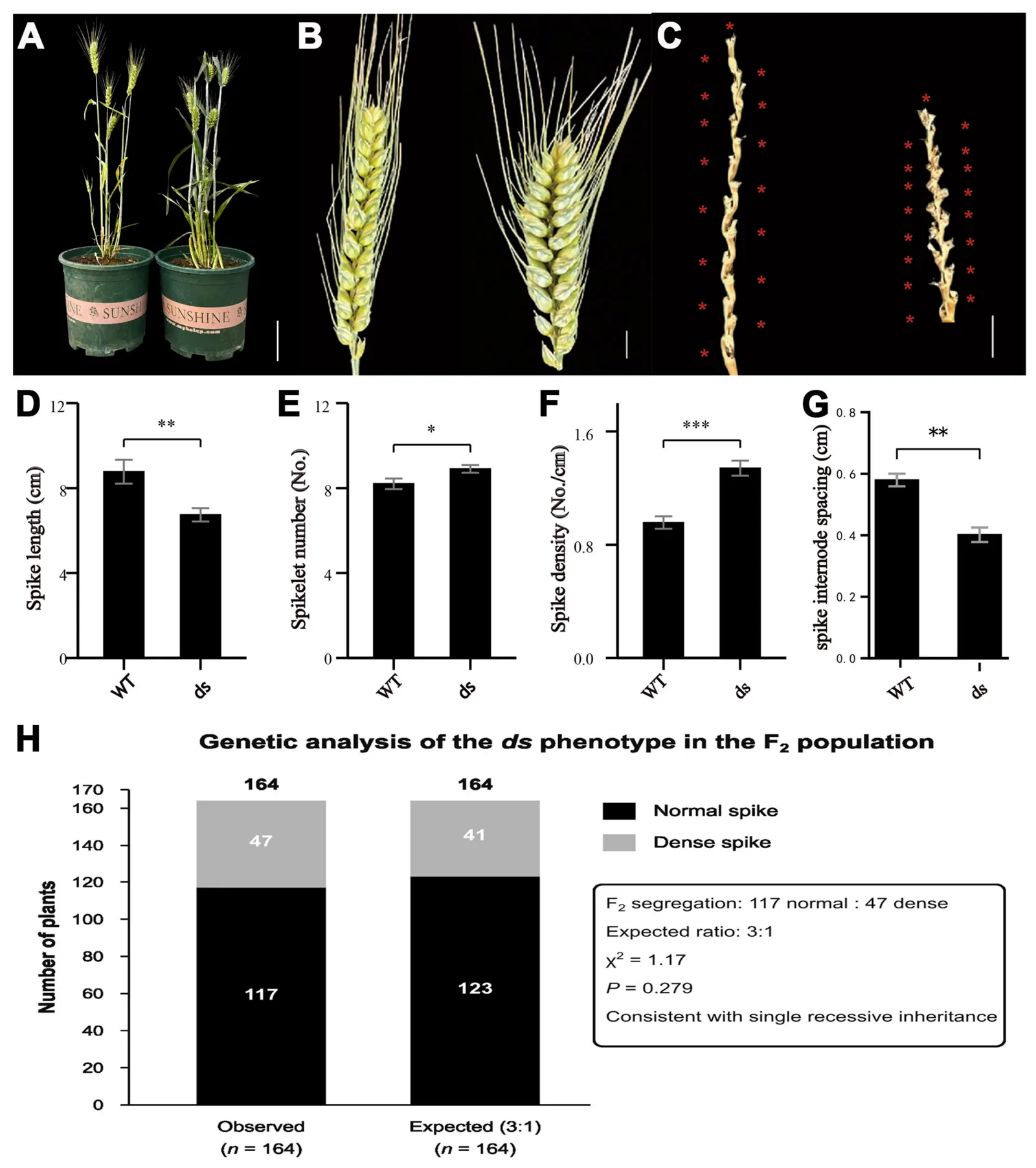
Phenotypic characterization and genetic analysis of the dense-spike mutant *ds* in wheat. (**A**) Whole-plant morphology of WT and *ds* plants at the adult stage. (**B**) Representative spikes of WT and *ds*. (**C**) Spike rachis morphology after removal of spikelets; red asterisks indicate spikelet attachment sites. (**D**–**G**) Quantitative comparison of spike length, spikelet number, spike density, and spike internode spacing between WT and *ds*. Bars represent mean ± SE (n = 15); asterisks indicate significant differences between WT and *ds* (* *p* < 0.05, ** *p* < 0.01, and *** *p* < 0.001). (**H**) Genetic analysis of the *ds* phenotype in the F_2_ population. The observed segregation fitted the expected 3:1 ratio for a single recessive nuclear gene.

The *ds* mutant exhibited a clearly distinguishable dense-spike phenotype compared with wild-type YN21 (WT) (Figure 1A–C). Although the overall plant architecture appeared largely comparable between WT and *ds*, the mutant exhibited a more compact spike with visibly reduced spacing between spikelets. After removal of spikelets, spikelet attachment sites were more closely arranged along the spike axis in *ds*, further confirming the compact spike phenotype (Figure 1C).

Quantitative analysis showed that spike length was significantly reduced in *ds* (7.38 ± 0.43 cm) compared with WT (8.70 ± 0.38 cm) (Figure 1D), whereas spikelet number was significantly increased in the mutant (8.70 ± 0.82 versus 7.80 ± 0.63) (Figure 1E). Consequently, spike density was significantly increased in *ds* (1.18 ± 0.13 spikelets cm^−1^ versus 0.90 ± 0.10 spikelets cm^−1^) (Figure 1F). The average spike internode spacing was significantly reduced in *ds* (0.42 ± 0.09 cm versus 0.58 ± 0.10 cm) (Figure 1G), indicating that shortened spike-axis internodes directly contributed to the dense-spike architecture.

Together, these results indicate that the *ds* phenotype is structurally associated with a shortened spike axis, reduced spike internode spacing, and increased spikelet number. Spike density was therefore used as a quantitative descriptor of spike compactness, rather than as the criterion for phenotype identification.

### 2.2. Genetic Analysis of the ds Phenotype

To determine the inheritance pattern of the dense-spike phenotype, *ds* was crossed with the wheat variety J22. All 16 F_1_ plants exhibited normal spike morphology, indicating that the ds phenotype is recessive. In the F_2_ population derived from a single F_1_ plant, 117 individuals showed normal spike morphology, whereas 47 displayed the dense-spike phenotype. This segregation fitted the expected 3:1 ratio for a single recessive nuclear gene (χ^2^ = 1.17, *p* = 0.279; Figure 1H), demonstrating that the field-visible dense-spike phenotype is controlled by a single recessive gene.

### 2.3. Genome-Wide Marker Screening and Linkage Validation Identify Chromosome 2B as the ds-Linked Region

**Figure 2:**
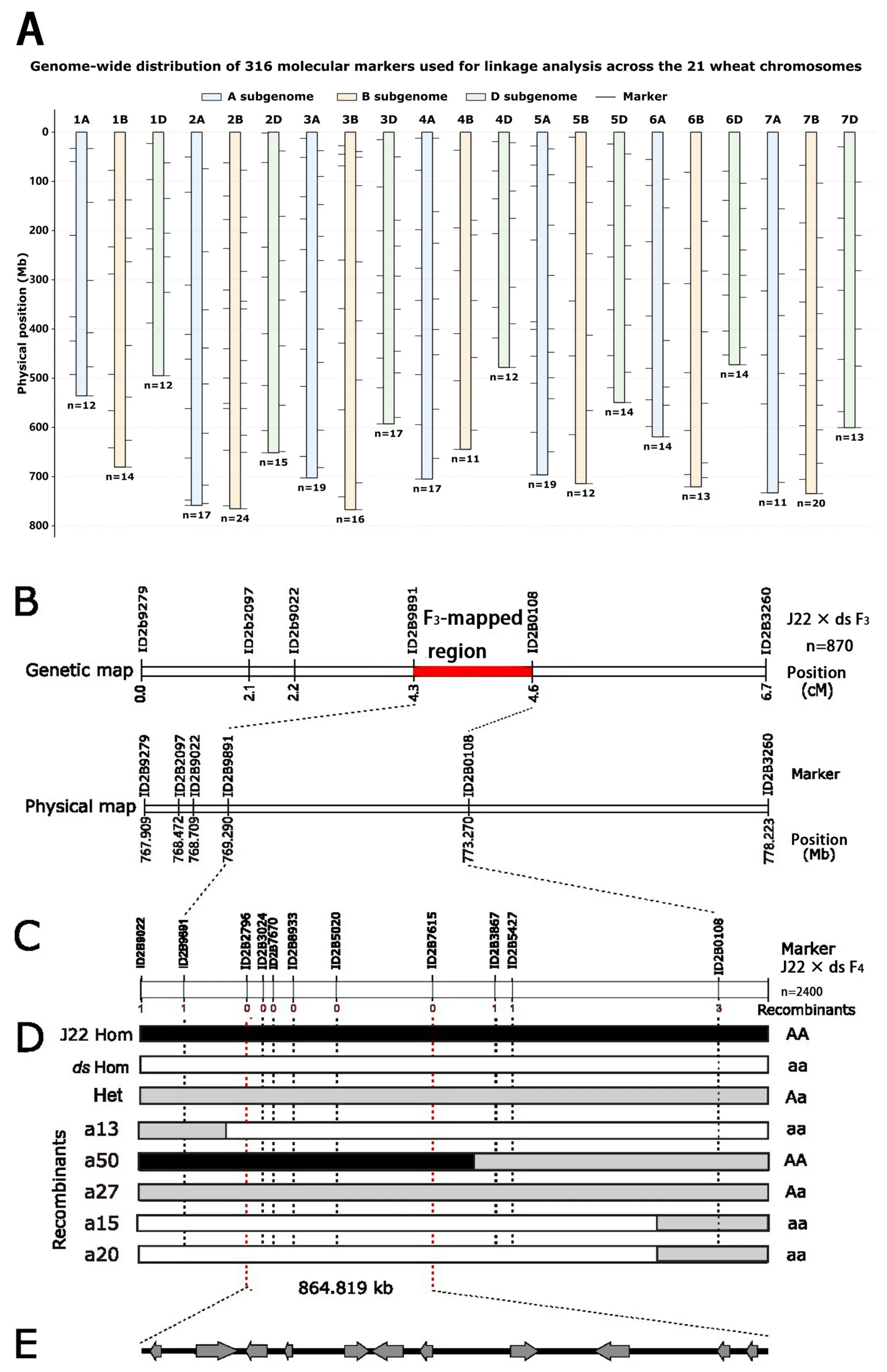
Identification and fine mapping of the genomic region underlying the recessive dense-spike mutant *ds*. (**A**) Genome-wide distribution of 316 polymorphic molecular markers used for linkage screening across the 21 wheat chromosomes. The A-, B-, and D-subgenome chromosomes are shown in different background shades. Short horizontal lines indicate marker positions, and the number of markers on each chromosome is shown. (**B**) Preliminary linkage validation and physical mapping of chromosome 2B markers. The markers ID2b9279 and ID2B3260 showed putative linkage with the *ds* phenotype and were used for enlarged-population validation. (**C**) Fine mapping using additional markers and recombinant individuals. The numbers below markers indicate recombinants detected between each marker and the *ds* locus. The red segment in panel B denotes the F_3_-mapped region, and the red dashed lines in panels C and D mark the boundaries of the final interval. (**D**) Graphical genotypes of representative recombinants used to delimit *ds*. Black, white, and gray bars represent homozygous J22, homozygous *ds*, and heterozygous regions, respectively. (**E**) Annotated genes within the final 864.819 kb fine-mapped interval between ID2B2796 and ID2B7615.

To identify molecular markers linked to the *ds* phenotype, 316 polymorphic markers, including SSR and InDel markers, distributed across all 21 wheat chromosomes were selected or newly developed based on the Chinese Spring reference genome sequence (IWGSC RefSeq v1.0 and v2.1) [27,28] and used for genome-wide preliminary linkage screening (Figure 2A). These markers provided broad coverage of the A, B, and D subgenomes and were used to screen 22 dense-spike individuals selected from a segregating population. Because the dense-spike phenotype was recessive, these individuals were expected to be homozygous for the *ds* allele, allowing markers potentially linked to the *ds* locus to be identified. The initial marker screen identified two chromosome 2B markers, ID2b9279 and ID2B3260, that showed putative linkage with the *ds* phenotype. These markers provided the first genetic evidence that the target locus was located on chromosome 2B. The 2B-linked region was therefore prioritized for enlarged-population validation and subsequent fine mapping.

Individual-level genotyping in an enlarged F_3_ population comprising 870 individuals further validated the linkage between the *ds* phenotype and the chromosome 2B region. The target region was first placed between flanking markers on the genetic and physical maps, and additional polymorphic markers were developed within this interval based on the IWGSC RefSeq v2.1 reference genome (Figure 2B). This step converted the preliminary genome-wide screening result into a chromosome 2B-linked candidate interval suitable for high-resolution mapping.

### 2.4. Fine Mapping of ds on Chromosome 2B

Fine mapping was performed using recombinant individuals identified from the above-mentioned F_3_ population, followed by further delimitation in a recombinant-derived F_4_ segregating population comprising 2400 plants. Newly developed markers within the chromosome 2B target region were genotyped and anchored to their physical positions, allowing the recombination breakpoints to be resolved (Figure 2C,D).

By integrating marker genotypes with progeny-tested phenotypes of key recombinants, the *ds* locus was delimited to an approximately 864.819 kb interval between ID2B2796 and ID2B7615 on chromosome 2B (Figure 2C–E). Gene annotation based on IWGSC RefSeq v2.1 identified 11 high-confidence genes within this interval, providing a focused candidate-gene set for further analysis (Table S1).

### 2.5. Candidate-Gene Prioritization Within the Fine-Mapped Interval

**Figure 3:**
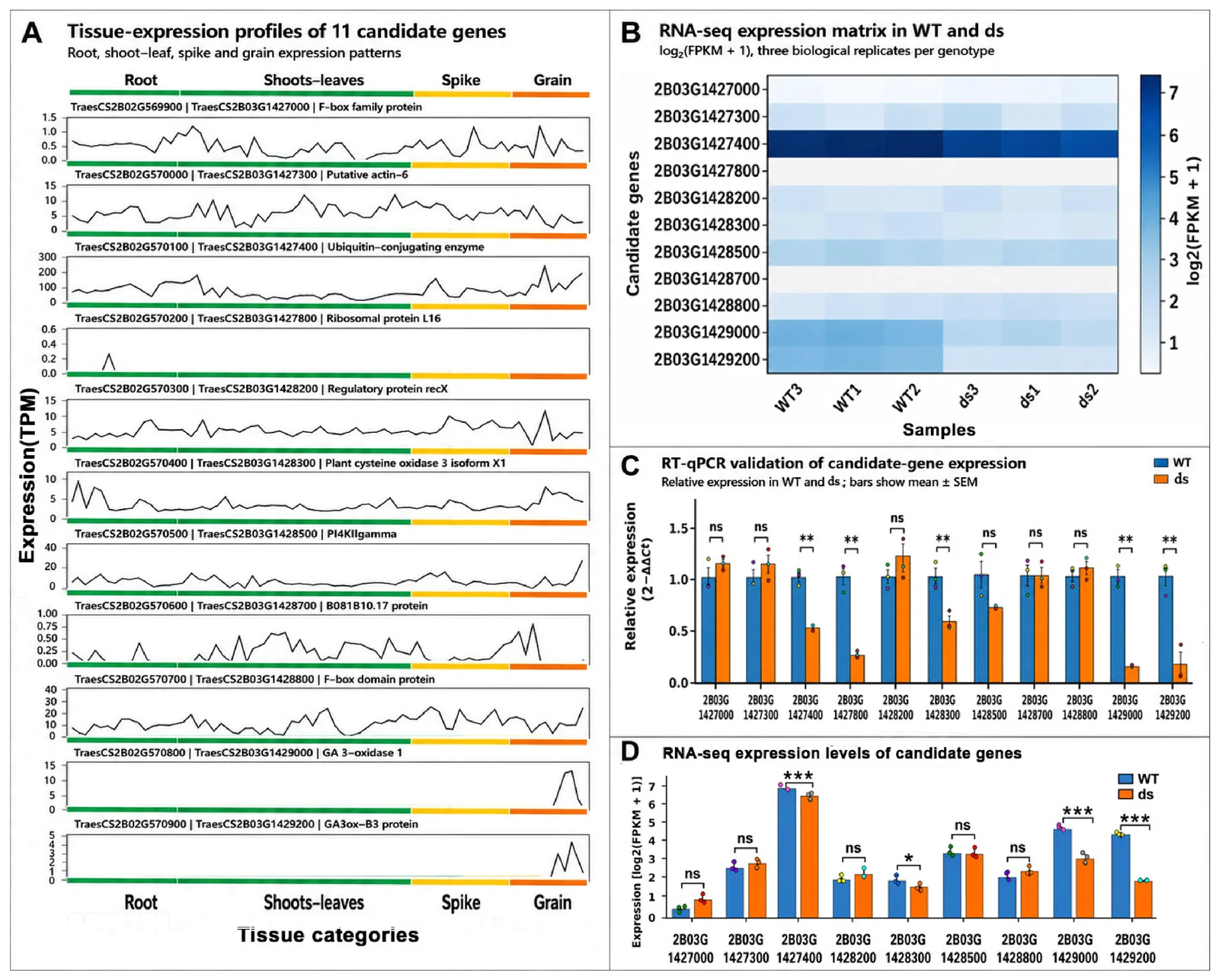
Expression profiling and validation of candidate genes within the fine-mapped *ds* interval. (**A**) Public tissue-expression profiles of the 11 high-confidence candidate genes located in the fine-mapped *ds* interval. Expression patterns are shown across root, shoot–leaf, spike, and grain tissues, and transcript abundance is presented as TPM. Colored bars below each profile indicate the corresponding tissue categories: green denotes root and shoot–leaf, yellow denotes spike, and orange denotes grain. The individual sample identities, tissues, and developmental stages grouped in these four categories are detailed in Figure S1. Gene identifiers from the previous and current wheat genome annotations are shown together, followed by the predicted gene annotation. (**B**) RNA-seq expression matrix of candidate genes in young spike tissues from WT-sib and *ds-*sib plants (spike tissues collected immediately after full spike emergence (Zadoks GS59)). Expression values from three biological replicates per genotype are shown as log_2_(FPKM + 1). (**C**) RT-qPCR validation of candidate-gene expression using independently collected backup young spike samples harvested at the same developmental stage and in the same experimental batch as the RNA-seq samples. Relative expression levels were calculated using the 2^−ΔΔCt^ method. Bars represent mean ± SEM, and dots indicate individual biological replicates. (**D**) Expression levels of the nine candidate genes for which comparable expression data were available in the published RNA-seq dataset. *TraesCS2B03G1427800* and *TraesCS2B03G1428700* were not included because corresponding expression values could not be retrieved from this dataset. Their omission from panel D should not be interpreted as evidence of absent expression. All 11 candidate genes were independently examined by RT-qPCR in panel C. Bars represent mean expression values based on three biological replicates; blue and orange bars denote WT-sib and *ds*-sib, respectively. Statistical significance was determined using Student’s *t*-test. ns, not significant; *, *p* < 0.05; **, *p* < 0.01; ***, *p* < 0.001.

Functional annotation of the 11 high-confidence genes in the final interval revealed diverse predicted functions, including F-box proteins, actin, ubiquitin-conjugating enzyme, ribosomal protein L16, regulatory protein *RecX*, plant cysteine oxidase, PI4KIIγ, and two adjacent *GA3OX-*family-related genes (Table S1; Figure 3A). All 11 genes were retained in the candidate-gene set and summarized in Table S1; therefore, the prioritization described below should not be interpreted as excluding other interval genes. Among these candidates, *TraesCS2B03G1429000* and *TraesCS2B03G1429200* were retained for further assessment because recent wheat gene-family annotation identifies them as *GA1OX1-B1*, a *GA3OX-*related *GA 1-*oxidase, and *GA3OX3-B1*, respectively [29].

Public tissue-expression profiles showed that the interval genes differed substantially in expression pattern and abundance across wheat tissues; the individual sample identities, tissues, and developmental stages represented in the four categories are provided in Figure S1 (Figure 3A). RNA-seq analysis of WT-sib and *ds*-sib young spike tissues further showed that *GA1OX1-B1* and *GA3OX3-B1* were expressed in the analyzed tissue and were reduced in *ds-*sib relative to WT-sib (Figure 3B,D). RT-qPCR using independently collected backup young spike samples from the same experimental batch and developmental stage supported the RNA-seq expression pattern and confirmed reduced expression of *TraesCS2B03G1429000* and *TraesCS2B03G1429200* in *ds-*sib (Figure 3C). These expression data provide supportive evidence for candidate prioritization but do not by themselves demonstrate a causal relationship between these genes and the ds phenotype.

To place the two interval genes in a family-wide context, published expression data for all seven *GA3OX*-related wheat genes were compiled across 70 tissues and developmental stages (Figure S2) [29,30,31]. The GA3OX2 A-, B-, and D-genome homoeologs showed broad expression across vegetative and reproductive tissues, whereas the GA3OX3 homoeologs and the B-genome-specific *GA1OX1-B1* displayed predominantly grain-associated profiles and low or undetectable expression in most publicly available spike samples. These public atlas data do not preclude genotype- or stage-specific expression in the young spikes analyzed here.

Taken together, the physical location of the 11 genes within the fine-mapped interval defines a focused candidate-gene set for *ds*. The two adjacent *GA3OX*-family-related genes are plausible, non-exclusive candidates because of their interval position, gene-family annotation, and reduced expression in the mutant. Nevertheless, because no consistently validated coding or regulatory mutation has yet been identified, the causative molecular lesion of *ds* remains unresolved and will require further validation.

### 2.6. Transcriptome Profiling Reveals Substantial Transcriptional Differences Between WT-sib and ds-sib

**Figure 4:**
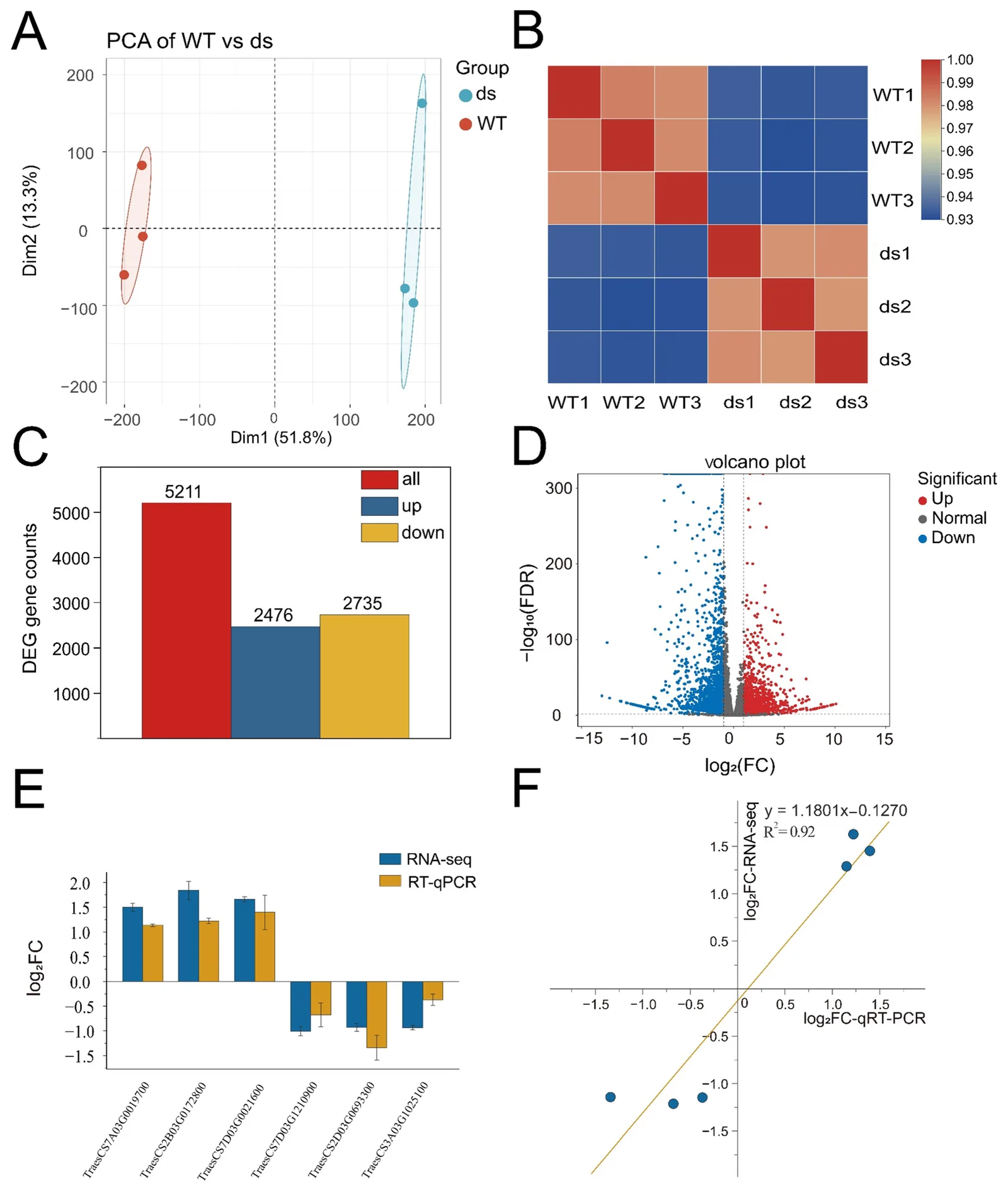
Overview and validation of RNA-seq analysis between WT-sib (WT1, WT2, WT3) and *ds-*sib (*ds*1, *ds*2, *ds*3) sibling segregants. (**A**) Principal component analysis of transcriptome data. (**B**) Sample-to-sample correlation heatmap. (**C**) Numbers of total, upregulated, and downregulated DEGs. (**D**) Volcano plot of DEGs. (**E**) RT-qPCR validation of selected DEGs. (**F**) Correlation between RNA-seq and RT-qPCR expression changes.

To explore molecular changes associated with the *ds* phenotype, RNA-seq analysis was performed using young spike tissues from WT-sib and *ds*-sib plants. Principal component analysis clearly separated the two sibling genotypes, with PC1 explaining 51.8% of the variance (Figure 4A). Pearson correlation analysis showed high consistency among biological replicates within each genotype (Figure 4B).

A total of 5211 differentially expressed genes (DEGs) were identified between WT-sib and *ds*-sib, including 2476 upregulated and 2735 downregulated genes in *ds-*sib (Figure 4C,D; Table S2). RT-qPCR validation of selected DEGs showed expression trends consistent with RNA-seq data, and the correlation between RNA-seq and RT-qPCR results was high (R^2^ = 0.92; Figure 4E,F), supporting the reliability of the transcriptome dataset. These results indicate that substantial transcriptional differences exist between WT-sib and *ds*-sib in developing spike tissues.

### 2.7. DEGs Are Enriched in Cell-Wall Organization, Carbohydrate Metabolism, Hormone-Related Processes, and Transcription-Factor Pathways

**Figure 5:**
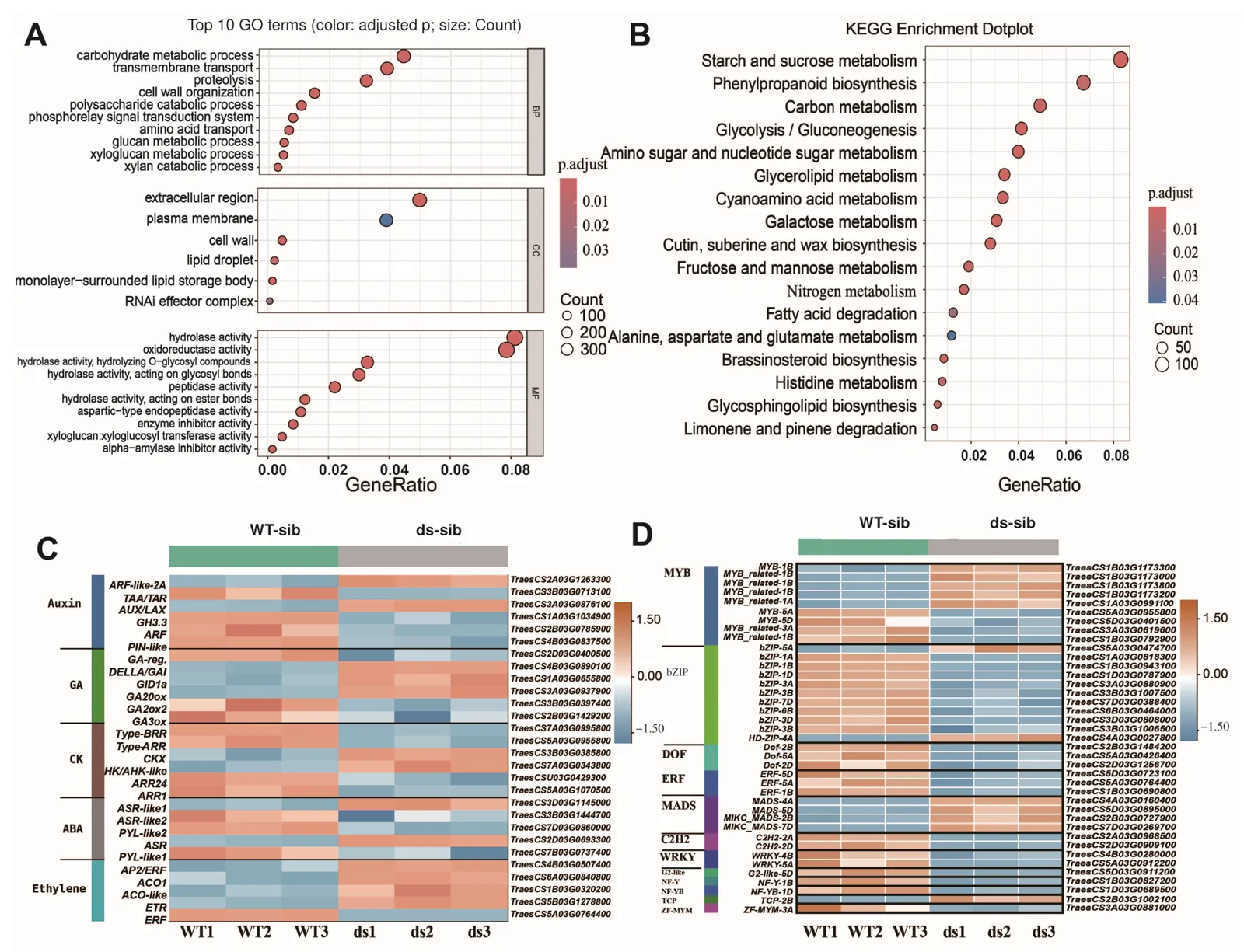
Functional enrichment and transcriptome profiling of differentially expressed genes between WT-sib and *ds*-sib. (**A**) GO enrichment analysis of DEGs. (**B**) KEGG enrichment analysis of DEGs. (**C**) Heatmap of selected hormone-related DEGs. (**D**) Heatmap of differentially expressed transcription factor genes.

GO enrichment analysis showed that DEGs were significantly enriched in biological processes and molecular functions related to carbohydrate metabolic process, transmembrane transport, proteolysis, cell-wall organization, hydrolase activity, and oxidoreductase activity (Figure 5A). KEGG enrichment analysis further highlighted starch and sucrose metabolism, phenylpropanoid biosynthesis, carbon metabolism, glycolysis/gluconeogenesis, amino sugar and nucleotide sugar metabolism, and brassinosteroid biosynthesis (Figure 5B). These enriched categories indicate that genes involved in basic metabolic and structural processes show altered expression between WT-sib and *ds*-sib.

Hormone-related DEGs were identified across multiple pathways, including auxin, GA, cytokinin, ABA, and ethylene pathways (Figure 5C; Table S3). In addition, differentially expressed transcription factors included members of the MYB, bZIP, DOF, ERF, MADS, C2H2, WRKY, G2-like, NF-Y, TCP, and ZF-MYM families (Figure 5D; Table S4). These results indicate that the *ds* mutation is accompanied by broad transcriptional reprogramming of hormone signaling, cell-wall metabolism, carbohydrate metabolism, and developmental regulators.

## 3. Discussion

### 3.1. ds Represents a Discrete Recessive Dense-Spike Mutant

Spike density is a quantitative index of spike architecture that integrates spike length, spikelet number, and rachis/spike-axis organization [6,7]. However, the EMS-induced *ds* mutant characterized in this study represents a discrete, field-visible dense-spike mutant phenotype rather than an arbitrary spike-density class. Quantitative values of spike density were used only to describe spike compactness and were not used to diagnose or define the phenotype. The classification used for genetic analysis was based on visible spike morphology, including compact spike architecture and reduced spikelet spacing. Consistent with this classification, all 16 F_1_ plants showed normal spike morphology, and the F_2_ population fitted a 3:1 segregation ratio, supporting control by a single recessive nuclear gene.

### 3.2. The ds Phenotype Is Mainly Determined by Reduced Spike-Axis Elongation

Classical and recent studies have shown that wheat and barley spike morphology can be altered either by modifying spikelet initiation and meristem determinacy or by changing longitudinal growth of the rachis/spike axis [1,3,8,9]. The *ds* mutant exhibits a moderate increase in spikelet number together with a pronounced reduction in spike length and internode spacing. This observation strongly suggests that the dense-spike phenotype of *ds* is primarily associated with defects in longitudinal growth of the spike axis rather than with spikelet initiation alone. Such a mechanism is consistent with previous reports showing that stem and inflorescence elongation in cereals and other plants is largely controlled by cell-elongation processes regulated by hormonal signals, especially gibberellins [14,15,17].

### 3.3. GA3OX-Family-Related Genes Are Plausible but Unconfirmed Candidates

Among the 11 high-confidence genes in the fine-mapped interval, *TraesCS2B03G1429000* and *TraesCS2B03G1429200* were retained as plausible but non-exclusive candidates. Recent functional characterization identified seven *GA3OX*-related homologues in bread wheat: the homoeologous GA3OX2 triad on chromosome group 3, the homoeologous GA3OX3 triad on chromosomes 2A, 2B, and 2D, and the B-genome-specific *GA1OX1-B1* [29]. The two genes in the mapped interval correspond to *GA1OX1-B1* (*TraesCS2B03G1429000*), a *GA3OX*-related GA 1-oxidase, and *GA3OX3-B1* (*TraesCS2B03G1429200*); thus, *GA3OX3-B1* has homoeologs on chromosomes 2A and 2D, whereas *GA1OX1-B1* is B-genome specific. The GA3OX2 triad was reported to be broadly expressed across vegetative and reproductive tissues, whereas the GA3OX3 homoeologs and *GA1OX1-B1* were predominantly expressed in developing grains [29]. In our dataset, the two chromosome-2B genes were nevertheless detected in young spikes and showed reduced expression in *ds*-sib relative to WT-sib. Their interval position and expression therefore justify retention for follow-up, but do not establish either gene as causal. *GA1OX1-B1* originated from a wheat-specific tandem duplication on chromosome 2B and lacks corresponding A- or D-genome homoeologs [29]. The family-wide public expression profiles (Figure S2) further showed low or undetectable expression of the GA3OX3 homoeologs and *GA1OX1-B1* in most spike samples, whereas both interval genes were detected in our stage-matched young-spike dataset. This difference may reflect genotype, precise developmental stage, or tissue-sampling resolution. Importantly, the reduced expression observed in *ds*-sib could also be a downstream consequence of altered spike development.

The underlying molecular lesion of *ds* remains unresolved. The absence of severe pleiotropic effects also warrants caution: broad defects might be expected if a central GA-biosynthetic activity were strongly disrupted, whereas homoeolog redundancy in hexaploid wheat and the distinct expression and biochemical functions of GA3OX3 and GA1OX1 family members could buffer or spatially restrict phenotypic effects [29]. Consequently, the lack of broad pleiotropy neither confirms nor excludes either interval gene. Because *ds* was derived from EMS mutagenesis, the phenotype may result from a coding, non-coding regulatory, intronic, or splice-site variant within the mapped interval that affects candidate-gene expression or function. The currently available expression evidence cannot distinguish whether reduced expression of the two *GA3OX*-family-related genes is causal or a downstream consequence of the *ds* mutation. Further work, including complete sequencing of the candidate interval, allele-specific expression analysis, GA measurement, exogenous GA response assays, and transgenic complementation or genome editing, will be required to determine the responsible gene and molecular mechanism.

Published mutant analyses provide additional context for the pleiotropy concern. Simultaneous disruption of the GA3OX2 homoeologs causes severe dwarfism, abnormal reproductive development, and infertility, whereas mutations affecting GA3OX3 and GA1OX1 predominantly alter grain GA concentrations and grain size and have comparatively moderate effects on plant height [29]. This contrast demonstrates substantial functional and spatial differentiation within the wheat *GA3OX*-related family. Homoeolog redundancy could potentially buffer variation in *GA3OX3-B1*, whereas the restricted expression and specialized biochemical activity of the B-genome-specific *GA1OX1-B1* could limit the breadth of its phenotypic effects. These possibilities remain hypotheses rather than evidence of causality.

### 3.4. Transcriptome Reprogramming Supports Hormone and Cell-Wall Involvement

Transcriptome analysis revealed extensive changes in hormone-related genes, cell-wall organization, carbohydrate metabolism, and transcription-factor families. Cell expansion depends on coordinated cell-wall loosening, polysaccharide remodeling, and wall-integrity regulation [22,23,25,26]. The differential expression and enrichment of genes related to cell-wall and carbohydrate metabolism may therefore be associated with reduced rachis internode spacing in *ds*. Similarly, changes in GA and auxin pathway genes suggest that hormone-associated transcriptional responses may accompany the *ds* phenotype rather than represent a single isolated molecular event, consistent with known interactions among GA, auxin, and other hormone pathways in plant architecture and organ growth [19,20,21]. These transcriptome results suggest that the *ds* mutation is associated with broad transcriptional changes during spike development. However, these changes likely represent downstream physiological responses rather than direct evidence of the causal gene or primary regulatory mechanism.

### 3.5. Remaining Questions and Future Validation

Although *TraesCS2B03G1429000* and *TraesCS2B03G1429200* remain plausible candidate genes, the current genetic and expression evidence does not establish causality. In forward-genetic studies, map-based cloning and sequencing can identify strong candidate genes, but independent functional assays remain important for demonstrating the relationship between genotype and phenotype [32]. Gene editing, genetic complementation, allelic analysis, GA-rescue experiments, and measurement of endogenous GA levels in developing spikes would help clarify whether and how local GA metabolism regulates wheat spike-axis elongation [15,17,29]. The family-wide expression profiles and published mutant phenotypes define the biological plausibility of these genes but do not substitute for identification and functional validation of the *ds* lesion.

## 4. Materials and Methods

### 4.1. Materials and Growth Conditions

The EMS-induced dense-spike mutant *ds* was obtained from an EMS-mutagenized population of the wheat cultivar YN21 generated in 2013. Briefly, dry seeds of YN21 were treated with 1.0% ethyl methanesulfonate (EMS; catalog no. M0880, Sigma-Aldrich, St. Louis, MO, USA) solution at room temperature for 18 h, thoroughly rinsed with running water, and then planted to generate the M1 population, following a modified wheat EMS mutagenesis procedure [33]. M1 plants were self-pollinated to produce M2 families, and plants showing increased spike compactness were selected in the M2 generation and further selfed for phenotypic confirmation. The stable dense-spike line was designated *ds*. The mutant has been stably maintained by selfing and shows a visually distinguishable compact spike phenotype. The parental line YN21, mutant *ds*, J22, and the F_1_, F_2_, F_3_, F_4_, and F_5_ segregating populations derived from the J22 × *ds* cross were planted in the experimental field of Yantai University, Yantai, Shandong Province, China. Field trials and population advancement were conducted across multiple growing seasons from 2013 to 2024. Plants were grown in row plots under local conventional field management. Each row was approximately 2.0 m long with 25 cm between rows, and plants were spaced approximately 10 cm apart within rows. Standard irrigation, fertilization, and pest-control practices were applied according to local wheat production recommendations. Relevant agronomic traits were investigated at plant maturity.

### 4.2. Phenotypic Identification and Quantitative Measurement of Spike Architecture

The dense-spike phenotype of *ds* was identified visually under field conditions based on compact spike architecture, shortened spike axis, and reduced spacing between spikelets. Spike-density values were not used as diagnostic thresholds for phenotype classification. Instead, spike length, spikelet number, spike density, and spike internode spacing were measured as quantitative descriptors of spike architecture. Following You et al. [34], spike density was calculated as the spikelet number per spike divided by spike length. Because spikelets were counted on one side of the spike in this study, spike density was calculated as SD = SSN/SL, where SSN represents the number of spikelets on one side and SL represents spike length.

### 4.3. Development of Genetic Populations and Inheritance Analysis

The dense-spike mutant *ds* was crossed with J22 to generate F_1_ plants. F_1_ plants were self-pollinated to produce the F_2_ population. The spike phenotype of F_1_ and F_2_ plants was recorded under field conditions, and segregation ratios were analyzed using χ^2^ tests. Progeny testing was used to confirm the genotype at the *ds* locus. For fine mapping, recombinant individuals were screened from F_3_ and F_4_ populations using polymorphic markers on chromosome 2B.

### 4.4. Genome-Wide Molecular-Marker Screening and Genotyping

Genomic DNA was extracted from young wheat leaves using the CTAB method [35,36]. A total of 316 polymorphic SSR/Indel molecular markers distributed across the 21 wheat chromosomes were selected or newly developed for genome-wide linkage screening. PCR amplification products were detected by polyacrylamide gel electrophoresis, and individual genotypes were determined based on polymorphic banding patterns. Markers showing linkage tendency with the *ds* phenotype were further validated in enlarged segregating populations.

### 4.5. Molecular Marker Development and Fine Mapping

Additional polymorphic markers were developed based on the Chinese Spring reference genome sequence (IWGSC RefSeq v1.0 and v2.1) [27,28]. Marker physical positions were determined according to the reference genome, and recombinant individuals were identified by genotyping markers in the chromosome 2B target region. The *ds*-linked interval was narrowed by integrating recombinant genotypes with progeny-tested phenotypes. Candidate genes within the final interval were annotated using IWGSC RefSeq v2.1.

### 4.6. RNA-Seq and Differential-Expression Analysis

At the heading stage, WT-sib and *ds*-sib plants were identified in the F_5_ segregating progeny obtained by self-pollination of a single F_4_ plant that was heterozygous across the *ds*-linked target interval. Individuals were genotyped with polymorphic markers spanning the target interval and assigned as WT-sib or *ds*-sib according to their target-region marker genotypes; these assignments were subsequently cross-checked against mature-spike phenotype. These materials were sibling segregants enriched for contrast at the mapped interval. Because genome-wide background uniformity was not tested, they are not described as near-isogenic lines. Young spike tissues were collected separately from WT-sib and *ds*-sib plants, with three biological replicates per genotype; each replicate consisted of pooled spike tissues from three individual plants. Samples were immediately frozen in liquid nitrogen and stored at −80 °C until RNA extraction. Total RNA was extracted using the FastPure Universal Plant Total RNA Isolation Kit (Vazyme, Nanjing, China) according to the manufacturer’s instructions. Transcriptome sequencing was performed using young spike tissues, with three biological replicates for each genotype. Sequencing libraries were constructed using standard Illumina protocols and sequenced on a paired-end 150 bp (PE150) platform (Illumina X). Raw reads were processed to remove adapters and low-quality reads using Trimmomatic v0.40 [37] and aligned to the wheat Chinese Spring reference genome RefSeq v2.1 using HISAT2 v2.2.3 [38]. Gene-level read counts were generated using featureCounts 2.1.1 for downstream analysis. RNA-seq quality and mapping statistics are provided in Table S6. Biological-replicate consistency was assessed using Pearson correlation analysis and principal component analysis (PCA). Raw read counts were used for differential-expression analysis with DESeq2 v1.52.0 in R v4.3.2 [39]. Differentially expressed genes were selected using fold change ≥ 2 and FDR < 0.01 as thresholds. GO and KEGG enrichment analyses were then performed using clusterProfiler v4.20.0 in R v4.3.2 with adjusted *p* < 0.05 as the significance threshold [40].

### 4.7. RNA Extraction and RT-qPCR

To validate RNA-seq results and candidate-gene expression patterns, RT-qPCR was performed using backup young spike samples that had been independently collected from WT-sib and *ds*-sib plants at the same developmental stage and in the same experimental batch as the RNA-seq samples (spike tissues collected immediately after full spike emergence (Zadoks GS59)). The RT-qPCR samples were distinct biological materials and were not aliquots of the RNA used for RNA-seq library construction. Total RNA was extracted using the FastPure Universal Plant Total RNA Isolation Kit (Vazyme, Nanjing, China) and reverse-transcribed using the HiScript II 1st Strand cDNA Synthesis Kit (Vazyme Biotech Co., Ltd., Nanjing, China). RT-qPCR reactions were performed using AceQ Universal SYBR qPCR Master Mix (Vazyme Biotech Co., Ltd., Nanjing, China) on a Roche LightCycler 480 Real-Time PCR System (Roche Diagnostics, Basel, Switzerland). Relative expression levels were calculated using the 2^−ΔΔCt^ method, with *GAPDH* as the internal reference gene. Three biological replicates were used per genotype. Gene-specific primers are listed in Table S5.

### 4.8. Public Tissue-Expression Analysis of Candidate Genes

Public wheat RNA-seq data from multiple tissues and developmental stages were obtained from WheatOmics and the Wheat Expression Browser [30,31] and used to examine expression patterns of candidate genes within the fine-mapped interval. Expression values were normalized as transcripts per million and grouped into root, shoot–leaf, spike, and grain categories. The individual sample identities, tissue types, and developmental stages included in each category are provided in Figure S1. The expression profiles were visualized using R. For the family-wide comparison, the seven *GA3OX*-related genes defined in a recent functional study [29] were examined using the same public datasets, and their expression profiles across 70 tissues and developmental stages are plotted in Figure S2.

## 5. Conclusions

In this study, we characterized *ds* as an EMS-induced recessive dense-spike mutant derived from the wheat cultivar YN21, with a shortened spike axis, reduced spike internode spacing, and increased spikelet number. Genetic analysis showed that the phenotype is controlled by a single recessive nuclear gene. Through genome-wide marker screening, enlarged-population linkage validation, and fine mapping, *ds* was delimited to an approximately 864.819 kb interval on chromosome 2B containing 11 high-confidence genes. Two adjacent *GA3OX*-family-related genes were retained as plausible, non-exclusive candidates based on their interval position, annotation, and reduced expression in the mutant; however, the current evidence does not establish causality, and no causal variant has yet been confirmed. Transcriptome analysis further revealed extensive changes in hormone-related pathways, cell-wall organization, carbohydrate metabolism, and transcription-factor regulation. These findings provide a reliable genetic basis for future cloning of *ds* and suggest that altered hormone- and growth-related regulation may contribute to dense-spike formation in wheat. The expanded family-wide expression comparison and published functional evidence support tissue- and family-member-specific roles for these genes but do not resolve the causal basis of *ds*.

## Data Availability

The RNA-sequencing read data generated in this study have been deposited in the NCBI Sequence Read Archive under BioProject accession PRJNA1511679. All other data are available in the article and its Supplementary Materials.

## Supplementary Materials

The following supporting information can be downloaded at https://www.mdpi.com/article/10.3390/plants15162470/s1, Figure S1: Tissue-expression profiles of candidate genes within the fine-mapped *ds* interval; Figure S2: Tissue-expression profiles of *GA3OX* family in wheat; Table S1: RNA-seq expression matrix and statistics of candidate genes within the fine-mapped *ds* interval; Table S2: RNA-seq expression matrix and differential expression statistics between NIL-WT and NIL-*ds*; Table S3: Selected hormone-related differentially expressed genes between NIL-WT and NIL-*ds*; Table S4: Differentially expressed transcription factor genes between NIL-WT and NIL-*ds*; Table S5: Primers used for genetic mapping, candidate-gene validation, and RT-qPCR analysis in this study; Table S6: RNA-seq quality and mapping statistics; Table S7: *GA3OX* genes in wheat.
