# Supplementary material for: Characterization and Fine Mapping of ds, a Recessive Dense-Spike Mutant Associated with Shortened Spike Axis and Increased Spikelet Number in Wheat
Source: Plants (Basel). 2026 Aug 14;15(16):2470. doi: 10.3390/plants15162470 (PMC13516082; doi:10.3390/plants15162470)
Supplement: Supplementary file 1 [file plants-15-02470-s001.zip › Figure S1. Tissue-expression profiles of candidate genes within the fine-mapped ds interval.pdf]

Figure S1. Expression patterns of candidate genes

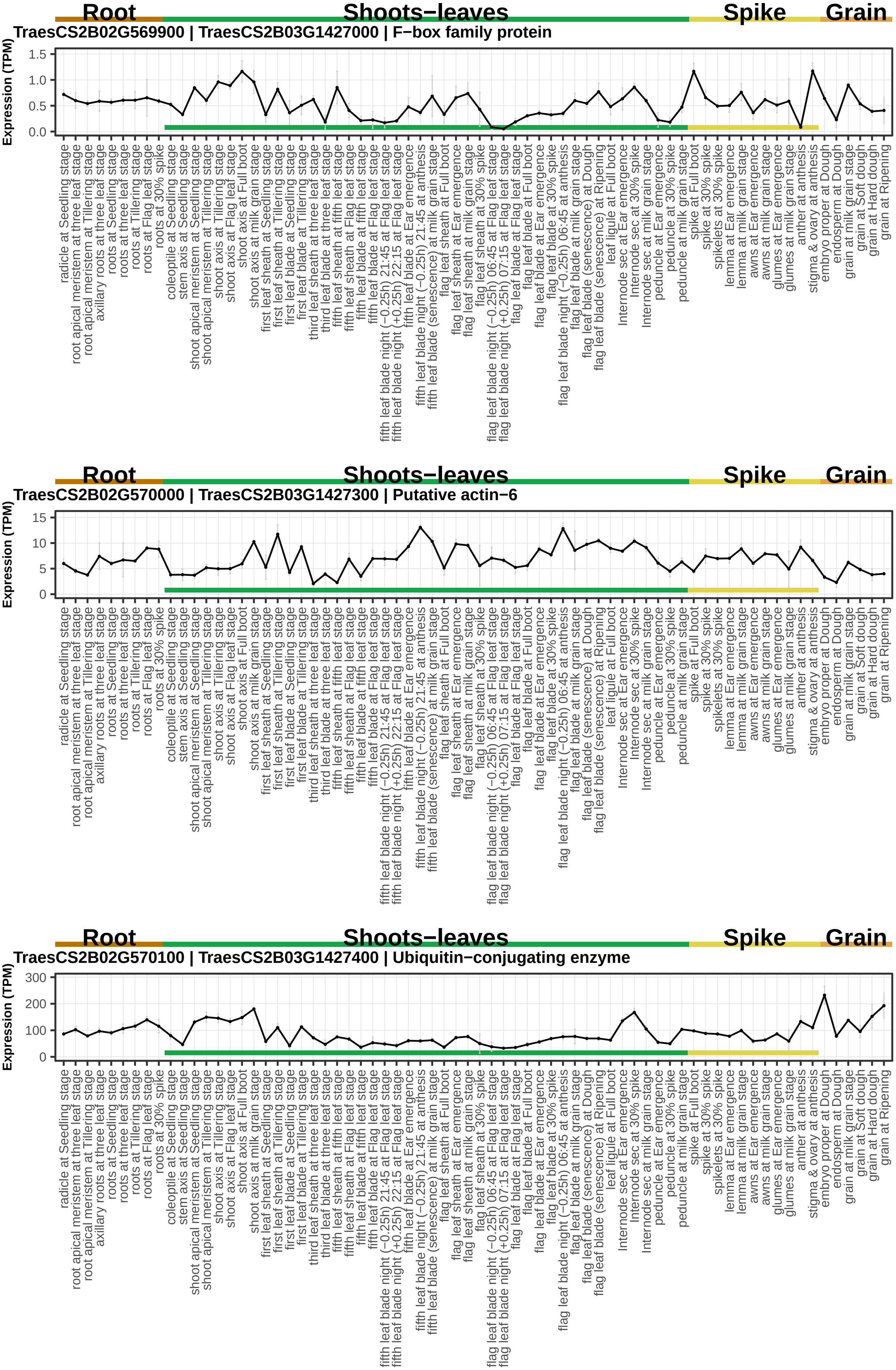

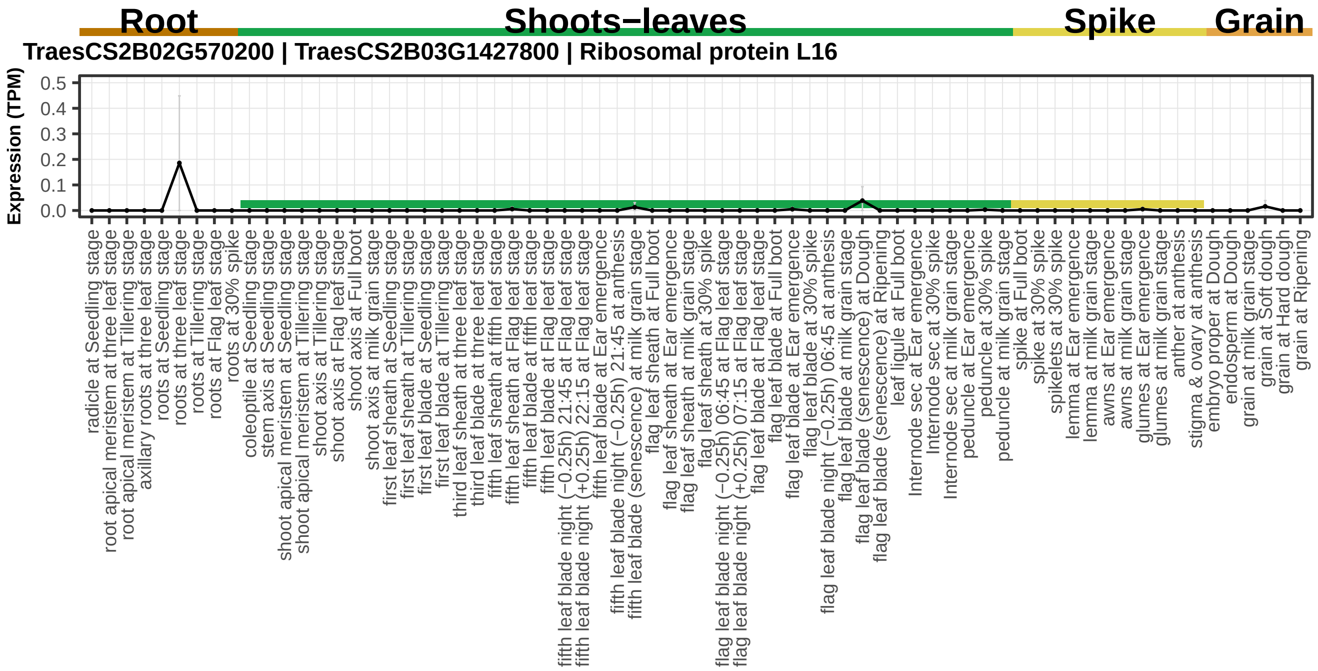
Root
Shoots−leaves
Spike
Grain
TraesCS2B02G570200 | TraesCS2B03G1427800 | Ribosomal protein L16
Expression (TPM)
0.5
0.4
0.3
0.2
0.1
0.0
radicle at Seedling stage
root apical meristem at three leaf stage
root apical meristem at Tillering stage
axillary roots at three leaf stage
roots at Seedling stage
roots at three leaf stage
roots at Tillering stage
roots at Flag leaf stage
roots at 30% spike
coleoptile at Seedling stage
stem axis at Seedling stage
shoot apical meristem at Seedling stage
shoot apical meristem at Tillering stage
shoot axis at Flag leaf stage
shoot axis at Full boot
shoot axis at milk grain stage
first leaf sheath at Seedling stage
first leaf sheath at Tillering stage
first leaf blade at Seedling stage
first leaf blade at Tillering stage
third leaf sheath at three leaf stage
third leaf blade at three leaf stage
fifth leaf sheath at fifth leaf stage
fifth leaf sheath at Flag leaf stage
fifth leaf blade at fifth leaf stage
fifth leaf blade at Flag leaf stage
fifth leaf blade night (−0.25h) 21:45 at Flag leaf stage
fifth leaf blade night (+0.25h) 22:15 at Flag leaf stage
fifth leaf blade at Ear emergence
fifth leaf blade night (−0.25h) 21:45 at anthesis
fifth leaf blade (senescence) at milk grain stage
flag leaf sheath at Full boot
flag leaf sheath at Ear emergence
flag leaf sheath at milk grain stage
flag leaf sheath at 30% spike
flag leaf blade night (−0.25h) 06:45 at Flag leaf stage
flag leaf blade night (+0.25h) 07:15 at Flag leaf stage
flag leaf blade at Flag leaf stage
flag leaf blade at Full boot
flag leaf blade at Ear emergence
flag leaf blade at 30% spike
flag leaf blade night (−0.25h) 06:45 at anthesis
flag leaf blade at milk grain stage
flag leaf blade (senescence) at Dough
flag leaf blade (senescence) at Ripening
leaf ligule at Full boot
Internode sec at Ear emergence
Internode sec at 30% spike
Internode sec at milk grain stage
peduncle at Ear emergence
peduncle at 30% spike
peduncle at milk grain stage
spike at Full boot
spike at 30% spike
spikelets at 30% spike
lemma at Ear emergence
lemma at milk grain stage
awns at Ear emergence
awns at milk grain stage
glumes at Ear emergence
glumes at milk grain stage
anther at anthesis
stigma & ovary at anthesis
embryo proper at Dough
endosperm at Dough
grain at milk grain stage
grain at Soft dough
grain at Hard dough
grain at Ripening

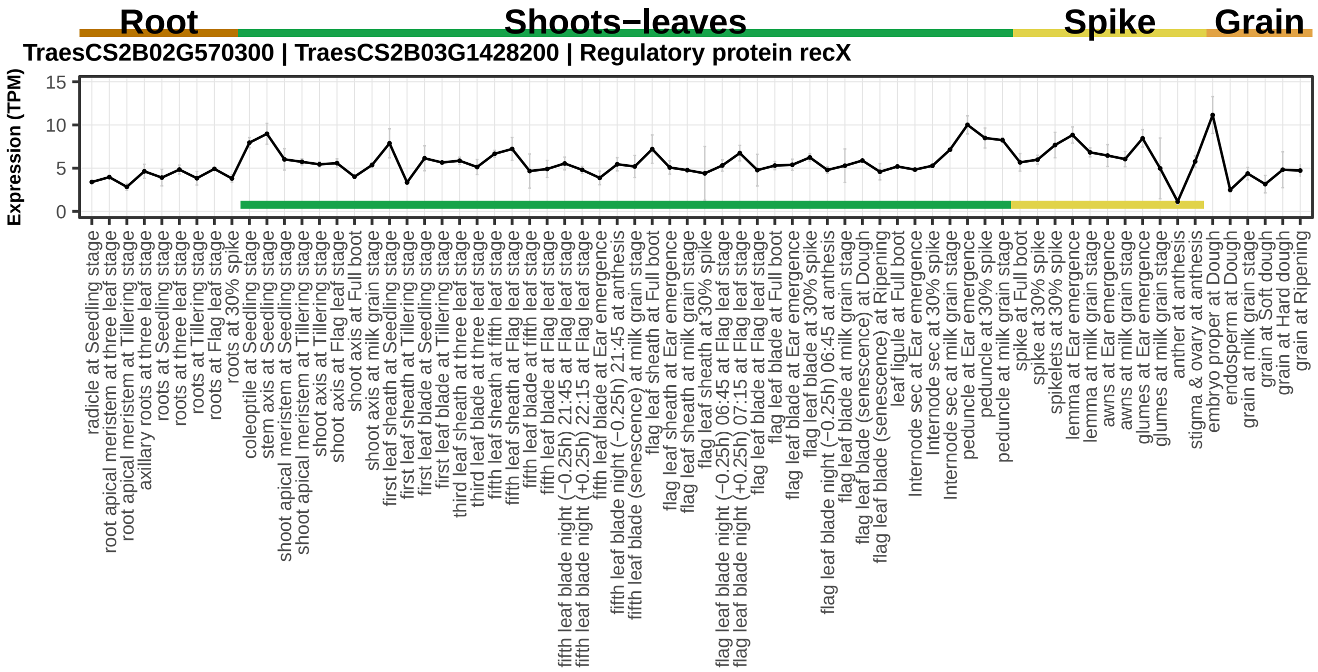
Root
Shoots−leaves
Spike
Grain
TraesCS2B02G570300 | TraesCS2B03G1428200 | Regulatory protein recX
Expression (TPM)
15
10
5
0
radicle at Seedling stage
root apical meristem at three leaf stage
root apical meristem at Tillering stage
axillary roots at three leaf stage
roots at Seedling stage
roots at three leaf stage
roots at Tillering stage
roots at Flag leaf stage
roots at 30% spike
coleoptile at Seedling stage
stem axis at Seedling stage
shoot apical meristem at Seedling stage
shoot apical meristem at Tillering stage
shoot axis at Flag leaf stage
shoot axis at Full boot
shoot axis at milk grain stage
first leaf sheath at Seedling stage
first leaf sheath at Tillering stage
first leaf blade at Seedling stage
first leaf blade at Tillering stage
third leaf sheath at three leaf stage
third leaf blade at three leaf stage
fifth leaf sheath at fifth leaf stage
fifth leaf sheath at Flag leaf stage
fifth leaf blade at fifth leaf stage
fifth leaf blade at Flag leaf stage
fifth leaf blade night (−0.25h) 21:45 at Flag leaf stage
fifth leaf blade night (+0.25h) 22:15 at Flag leaf stage
fifth leaf blade at Ear emergence
fifth leaf blade night (−0.25h) 21:45 at anthesis
fifth leaf blade (senescence) at milk grain stage
flag leaf sheath at Full boot
flag leaf sheath at Ear emergence
flag leaf sheath at milk grain stage
flag leaf sheath at 30% spike
flag leaf blade night (−0.25h) 06:45 at Flag leaf stage
flag leaf blade night (+0.25h) 07:15 at Flag leaf stage
flag leaf blade at Flag leaf stage
flag leaf blade at Full boot
flag leaf blade at Ear emergence
flag leaf blade at 30% spike
flag leaf blade night (−0.25h) 06:45 at anthesis
flag leaf blade at milk grain stage
flag leaf blade (senescence) at Dough
flag leaf blade (senescence) at Ripening
leaf ligule at Full boot
Internode sec at Ear emergence
Internode sec at 30% spike
Internode sec at milk grain stage
peduncle at Ear emergence
peduncle at 30% spike
peduncle at milk grain stage
spike at Full boot
spike at 30% spike
spikelets at 30% spike
lemma at Ear emergence
lemma at milk grain stage
awns at Ear emergence
awns at milk grain stage
glumes at Ear emergence
glumes at milk grain stage
anther at anthesis
stigma & ovary at anthesis
embryo proper at Dough
endosperm at Dough
grain at milk grain stage
grain at Soft dough
grain at Hard dough
grain at Ripening

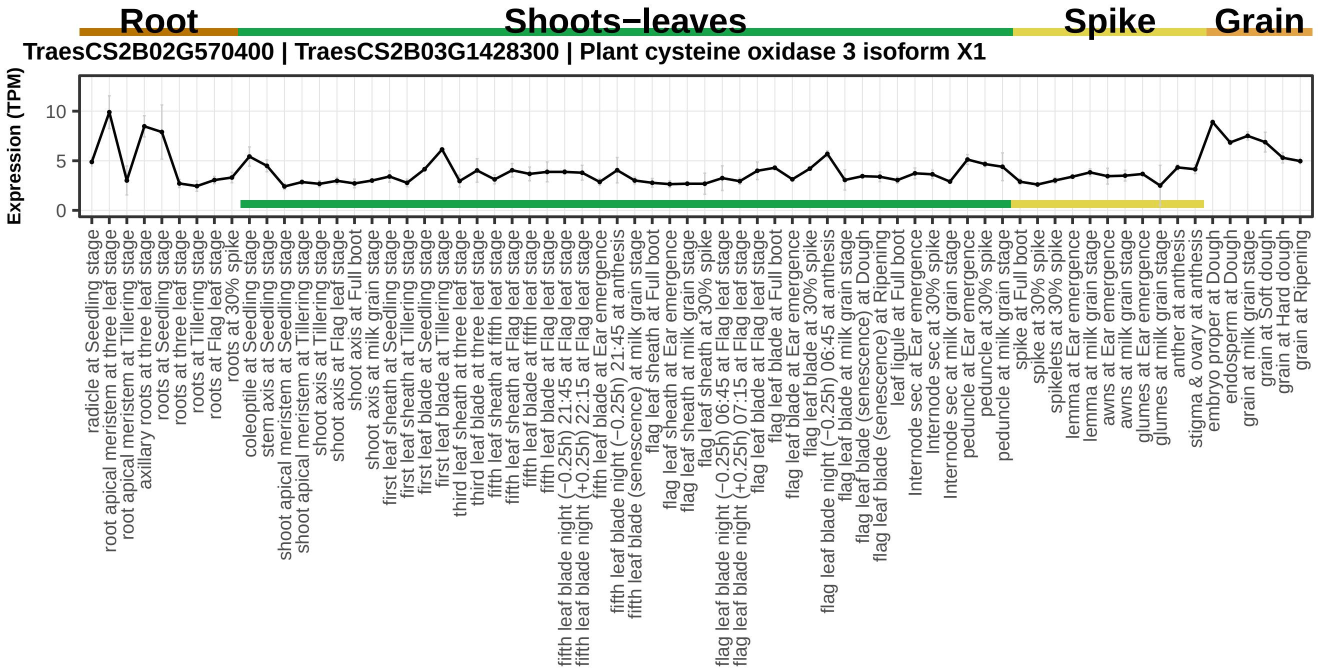
Root
Shoots−leaves
Spike
Grain
TraesCS2B02G570400 | TraesCS2B03G1428300 | Plant cysteine oxidase 3 isoform X1
Expression (TPM)
10
5
0
radicle at Seedling stage
root apical meristem at three leaf stage
root apical meristem at Tillering stage
axillary roots at three leaf stage
roots at Seedling stage
roots at three leaf stage
roots at Tillering stage
roots at Flag leaf stage
roots at 30% spike
coleoptile at Seedling stage
stem axis at Seedling stage
shoot apical meristem at Seedling stage
shoot apical meristem at Tillering stage
shoot axis at Flag leaf stage
shoot axis at Full boot
shoot axis at milk grain stage
first leaf sheath at Seedling stage
first leaf sheath at Tillering stage
first leaf blade at Seedling stage
first leaf blade at Tillering stage
third leaf sheath at three leaf stage
third leaf blade at three leaf stage
fifth leaf sheath at fifth leaf stage
fifth leaf sheath at Flag leaf stage
fifth leaf blade at fifth leaf stage
fifth leaf blade at Flag leaf stage
fifth leaf blade night (−0.25h) 21:45 at Flag leaf stage
fifth leaf blade night (+0.25h) 22:15 at Flag leaf stage
fifth leaf blade at Ear emergence
fifth leaf blade night (−0.25h) 21:45 at anthesis
fifth leaf blade (senescence) at milk grain stage
flag leaf sheath at Full boot
flag leaf sheath at Ear emergence
flag leaf sheath at milk grain stage
flag leaf sheath at 30% spike
flag leaf blade night (−0.25h) 06:45 at Flag leaf stage
flag leaf blade night (+0.25h) 07:15 at Flag leaf stage
flag leaf blade at Flag leaf stage
flag leaf blade at Full boot
flag leaf blade at Ear emergence
flag leaf blade at 30% spike
flag leaf blade night (−0.25h) 06:45 at anthesis
flag leaf blade at milk grain stage
flag leaf blade (senescence) at Dough
flag leaf blade (senescence) at Ripening
leaf ligule at Full boot
Internode sec at Ear emergence
Internode sec at 30% spike
Internode sec at milk grain stage
peduncle at Ear emergence
peduncle at 30% spike
peduncle at milk grain stage
spike at Full boot
spike at 30% spike
spikelets at 30% spike
lemma at Ear emergence
lemma at milk grain stage
awns at Ear emergence
awns at milk grain stage
glumes at Ear emergence
glumes at milk grain stage
anther at anthesis
stigma & ovary at anthesis
embryo proper at Dough
endosperm at Dough
grain at milk grain stage
grain at Soft dough
grain at Hard dough
grain at Ripening

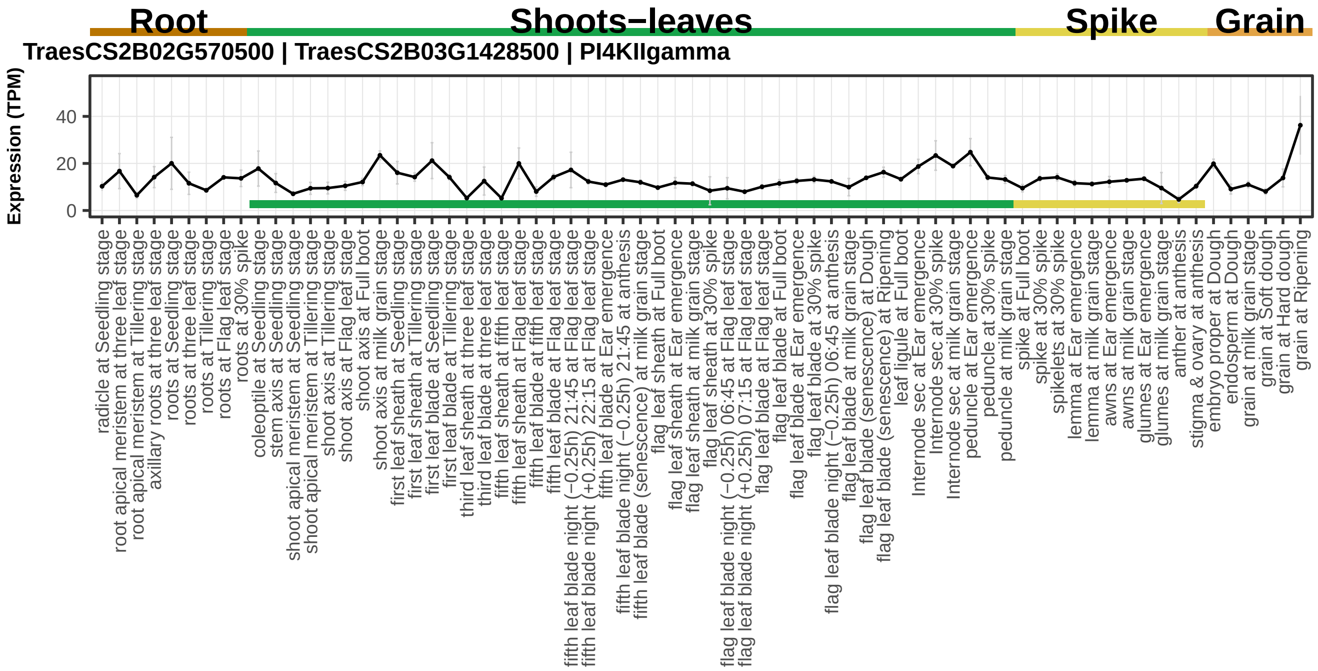
Root
Shoots−leaves
Spike
Grain
TraesCS2B02G570500 | TraesCS2B03G1428500 | PI4KIIgamma
Expression (TPM)
0
20
40
radicle at Seedling stage
root apical meristem at three leaf stage
root apical meristem at Tillering stage
axillary roots at three leaf stage
roots at Seedling stage
roots at three leaf stage
roots at Tillering stage
roots at Flag leaf stage
roots at 30% spike
coleoptile at Seedling stage
stem axis at Seedling stage
shoot apical meristem at Seedling stage
shoot apical meristem at Tillering stage
shoot axis at Tillering stage
shoot axis at Flag leaf stage
shoot axis at Full boot
shoot axis at milk grain stage
first leaf sheath at Seedling stage
first leaf sheath at Tillering stage
first leaf blade at Seedling stage
first leaf blade at Tillering stage
third leaf sheath at three leaf stage
third leaf blade at three leaf stage
fifth leaf sheath at fifth leaf stage
fifth leaf sheath at Flag leaf stage
fifth leaf blade at fifth leaf stage
fifth leaf blade at Flag leaf stage
fifth leaf blade night (−0.25h) 21:45 at Flag leaf stage
fifth leaf blade night (+0.25h) 22:15 at Flag leaf stage
fifth leaf blade at Ear emergence
fifth leaf blade night (−0.25h) 21:45 at anthesis
fifth leaf blade (senescence) at milk grain stage
flag leaf sheath at Full boot
flag leaf sheath at Ear emergence
flag leaf sheath at milk grain stage
flag leaf sheath at 30% spike
flag leaf blade night (−0.25h) 06:45 at Flag leaf stage
flag leaf blade night (+0.25h) 07:15 at Flag leaf stage
flag leaf blade at Flag leaf stage
flag leaf blade at Full boot
flag leaf blade at Ear emergence
flag leaf blade at 30% spike
flag leaf blade night (−0.25h) 06:45 at anthesis
flag leaf blade at milk grain stage
flag leaf blade (senescence) at Dough
flag leaf blade (senescence) at Ripening
leaf ligule at Full boot
Internode sec at Ear emergence
Internode sec at 30% spike
Internode sec at milk grain stage
peduncle at Ear emergence
peduncle at 30% spike
peduncle at milk grain stage
spike at Full boot
spike at 30% spike
spikelets at 30% spike
lemma at Ear emergence
lemma at milk grain stage
awns at Ear emergence
awns at milk grain stage
glumes at Ear emergence
glumes at milk grain stage
anther at anthesis
stigma & ovary at anthesis
embryo proper at Dough
endosperm at Dough
grain at milk grain stage
grain at Soft dough
grain at Hard dough
grain at Ripening

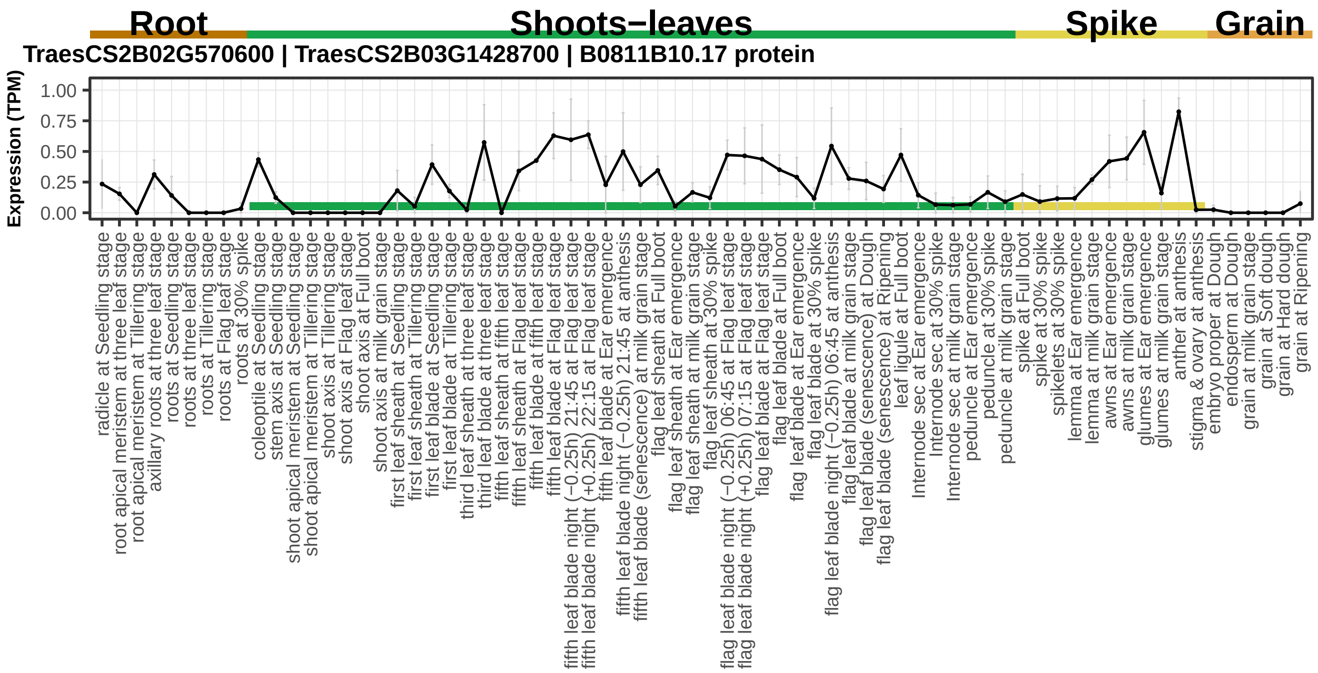
Root
Shoots−leaves
Spike
Grain
TraesCS2B02G570600 | TraesCS2B03G1428700 | B0811B10.17 protein
Expression (TPM)
0.00
0.25
0.50
0.75
1.00
radicle at Seedling stage
root apical meristem at three leaf stage
root apical meristem at Tillering stage
axillary roots at three leaf stage
roots at Seedling stage
roots at three leaf stage
roots at Tillering stage
roots at Flag leaf stage
roots at 30% spike
coleoptile at Seedling stage
stem axis at Seedling stage
shoot apical meristem at Seedling stage
shoot apical meristem at Tillering stage
shoot axis at Tillering stage
shoot axis at Flag leaf stage
shoot axis at Full boot
shoot axis at milk grain stage
first leaf sheath at Seedling stage
first leaf sheath at Tillering stage
first leaf blade at Seedling stage
first leaf blade at Tillering stage
third leaf sheath at three leaf stage
third leaf blade at three leaf stage
fifth leaf sheath at fifth leaf stage
fifth leaf sheath at Flag leaf stage
fifth leaf blade at fifth leaf stage
fifth leaf blade at Flag leaf stage
fifth leaf blade night (−0.25h) 21:45 at Flag leaf stage
fifth leaf blade night (+0.25h) 22:15 at Flag leaf stage
fifth leaf blade at Ear emergence
fifth leaf blade night (−0.25h) 21:45 at anthesis
fifth leaf blade (senescence) at milk grain stage
flag leaf sheath at Full boot
flag leaf sheath at Ear emergence
flag leaf sheath at milk grain stage
flag leaf sheath at 30% spike
flag leaf blade night (−0.25h) 06:45 at Flag leaf stage
flag leaf blade night (+0.25h) 07:15 at Flag leaf stage
flag leaf blade at Flag leaf stage
flag leaf blade at Full boot
flag leaf blade at Ear emergence
flag leaf blade at 30% spike
flag leaf blade night (−0.25h) 06:45 at anthesis
flag leaf blade at milk grain stage
flag leaf blade (senescence) at Dough
flag leaf blade (senescence) at Ripening
leaf ligule at Full boot
Internode sec at Ear emergence
Internode sec at 30% spike
Internode sec at milk grain stage
peduncle at Ear emergence
peduncle at 30% spike
peduncle at milk grain stage
spike at Full boot
spike at 30% spike
spikelets at 30% spike
lemma at Ear emergence
lemma at milk grain stage
awns at Ear emergence
awns at milk grain stage
glumes at Ear emergence
glumes at milk grain stage
anther at anthesis
stigma & ovary at anthesis
embryo proper at Dough
endosperm at Dough
grain at milk grain stage
grain at Soft dough
grain at Hard dough
grain at Ripening

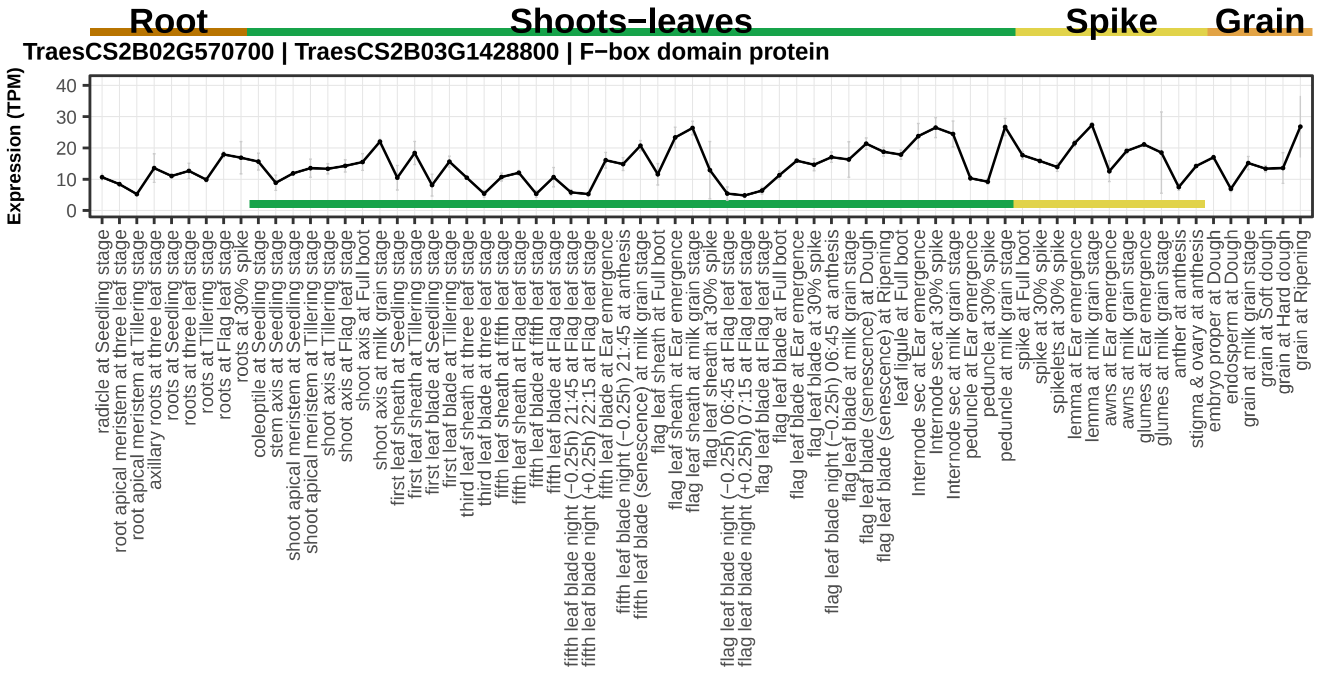
Root
Shoots−leaves
Spike
Grain
TraesCS2B02G570700 | TraesCS2B03G1428800 | F−box domain protein
Expression (TPM)
0
10
20
30
40
radicle at Seedling stage
root apical meristem at three leaf stage
root apical meristem at Tillering stage
axillary roots at three leaf stage
roots at Seedling stage
roots at three leaf stage
roots at Tillering stage
roots at Flag leaf stage
roots at 30% spike
coleoptile at Seedling stage
stem axis at Seedling stage
shoot apical meristem at Seedling stage
shoot apical meristem at Tillering stage
shoot axis at Tillering stage
shoot axis at Flag leaf stage
shoot axis at Full boot
shoot axis at milk grain stage
first leaf sheath at Seedling stage
first leaf sheath at Tillering stage
first leaf blade at Seedling stage
first leaf blade at Tillering stage
third leaf sheath at three leaf stage
third leaf blade at three leaf stage
fifth leaf sheath at fifth leaf stage
fifth leaf sheath at Flag leaf stage
fifth leaf blade at fifth leaf stage
fifth leaf blade at Flag leaf stage
fifth leaf blade night (−0.25h) 21:45 at Flag leaf stage
fifth leaf blade night (+0.25h) 22:15 at Flag leaf stage
fifth leaf blade at Ear emergence
fifth leaf blade night (−0.25h) 21:45 at anthesis
fifth leaf blade (senescence) at milk grain stage
flag leaf sheath at Full boot
flag leaf sheath at Ear emergence
flag leaf sheath at milk grain stage
flag leaf sheath at 30% spike
flag leaf blade night (−0.25h) 06:45 at Flag leaf stage
flag leaf blade night (+0.25h) 07:15 at Flag leaf stage
flag leaf blade at Flag leaf stage
flag leaf blade at Full boot
flag leaf blade at Ear emergence
flag leaf blade at 30% spike
flag leaf blade night (−0.25h) 06:45 at anthesis
flag leaf blade at milk grain stage
flag leaf blade (senescence) at Dough
flag leaf blade (senescence) at Ripening
leaf ligule at Full boot
Internode sec at Ear emergence
Internode sec at 30% spike
Internode sec at milk grain stage
peduncle at Ear emergence
peduncle at 30% spike
peduncle at milk grain stage
spike at Full boot
spike at 30% spike
spikelets at 30% spike
lemma at Ear emergence
lemma at milk grain stage
awns at Ear emergence
awns at milk grain stage
glumes at Ear emergence
glumes at milk grain stage
anther at anthesis
stigma & ovary at anthesis
embryo proper at Dough
endosperm at Dough
grain at milk grain stage
grain at Soft dough
grain at Hard dough
grain at Ripening

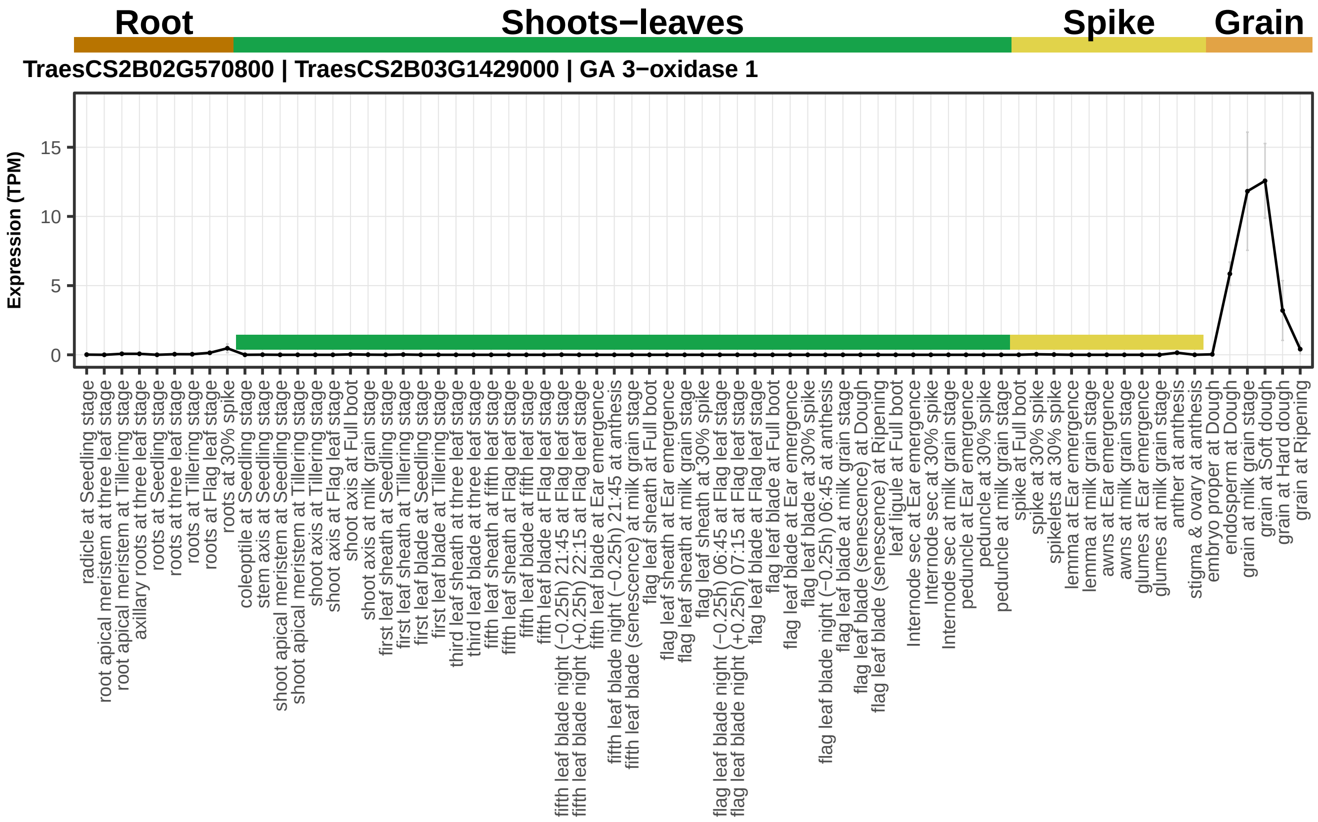
Root
Shoots−leaves
Spike
Grain
TraesCS2B02G570800 | TraesCS2B03G1429000 | GA 3−oxidase 1
Expression (TPM)
0
5
10
15
radicle at Seedling stage
root apical meristem at three leaf stage
root apical meristem at Tillering stage
axillary roots at three leaf stage
roots at Seedling stage
roots at three leaf stage
roots at Tillering stage
roots at Flag leaf stage
roots at 30% spike
coleoptile at Seedling stage
stem axis at Seedling stage
shoot apical meristem at Seedling stage
shoot apical meristem at Tillering stage
shoot axis at Tillering stage
shoot axis at Flag leaf stage
shoot axis at Full boot
shoot axis at milk grain stage
first leaf sheath at Seedling stage
first leaf sheath at Tillering stage
first leaf blade at Seedling stage
first leaf blade at Tillering stage
third leaf sheath at three leaf stage
third leaf blade at three leaf stage
fifth leaf sheath at fifth leaf stage
fifth leaf sheath at Flag leaf stage
fifth leaf blade at fifth leaf stage
fifth leaf blade at Flag leaf stage
fifth leaf blade night (−0.25h) 21:45 at Flag leaf stage
fifth leaf blade night (+0.25h) 22:15 at Flag leaf stage
fifth leaf blade at Ear emergence
fifth leaf blade night (−0.25h) 21:45 at anthesis
fifth leaf blade (senescence) at milk grain stage
flag leaf sheath at Full boot
flag leaf sheath at Ear emergence
flag leaf sheath at milk grain stage
flag leaf sheath at 30% spike
flag leaf blade night (−0.25h) 06:45 at Flag leaf stage
flag leaf blade night (+0.25h) 07:15 at Flag leaf stage
flag leaf blade at Flag leaf stage
flag leaf blade at Full boot
flag leaf blade at Ear emergence
flag leaf blade at 30% spike
flag leaf blade night (−0.25h) 06:45 at anthesis
flag leaf blade at milk grain stage
flag leaf blade (senescence) at Dough
flag leaf blade (senescence) at Ripening
leaf ligule at Full boot
Internode sec at Ear emergence
Internode sec at 30% spike
Internode sec at milk grain stage
peduncle at Ear emergence
peduncle at 30% spike
peduncle at milk grain stage
spike at Full boot
spike at 30% spike
spikelets at 30% spike
lemma at Ear emergence
lemma at milk grain stage
awns at Ear emergence
awns at milk grain stage
glumes at Ear emergence
glumes at milk grain stage
anther at anthesis
stigma & ovary at anthesis
embryo proper at Dough
endosperm at Dough
grain at milk grain stage
grain at Soft dough
grain at Hard dough
grain at Ripening

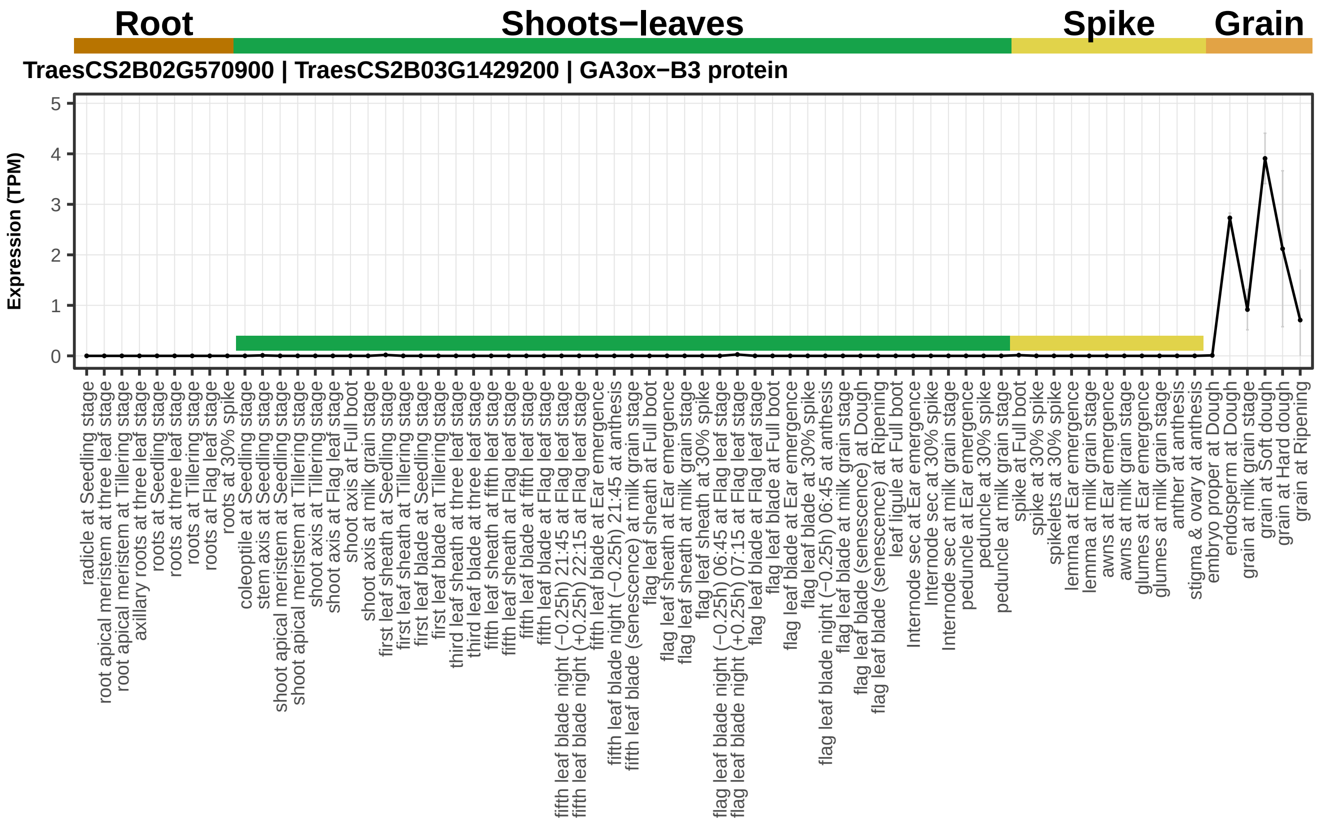
Root
Shoots−leaves
Spike
Grain
TraesCS2B02G570900 | TraesCS2B03G1429200 | GA3ox−B3 protein
Expression (TPM)
0
1
2
3
4
5
radicle at Seedling stage
root apical meristem at three leaf stage
root apical meristem at Tillering stage
axillary roots at three leaf stage
roots at Seedling stage
roots at three leaf stage
roots at Tillering stage
roots at Flag leaf stage
roots at 30% spike
coleoptile at Seedling stage
stem axis at Seedling stage
shoot apical meristem at Seedling stage
shoot apical meristem at Tillering stage
shoot axis at Tillering stage
shoot axis at Flag leaf stage
shoot axis at Full boot
shoot axis at milk grain stage
first leaf sheath at Seedling stage
first leaf sheath at Tillering stage
first leaf blade at Seedling stage
first leaf blade at Tillering stage
third leaf sheath at three leaf stage
third leaf blade at three leaf stage
fifth leaf sheath at fifth leaf stage
fifth leaf sheath at Flag leaf stage
fifth leaf blade at fifth leaf stage
fifth leaf blade at Flag leaf stage
fifth leaf blade night (−0.25h) 21:45 at Flag leaf stage
fifth leaf blade night (+0.25h) 22:15 at Flag leaf stage
fifth leaf blade at Ear emergence
fifth leaf blade night (−0.25h) 21:45 at anthesis
fifth leaf blade (senescence) at milk grain stage
flag leaf sheath at Full boot
flag leaf sheath at Ear emergence
flag leaf sheath at milk grain stage
flag leaf sheath at 30% spike
flag leaf blade night (−0.25h) 06:45 at Flag leaf stage
flag leaf blade night (+0.25h) 07:15 at Flag leaf stage
flag leaf blade at Flag leaf stage
flag leaf blade at Full boot
flag leaf blade at Ear emergence
flag leaf blade at 30% spike
flag leaf blade night (−0.25h) 06:45 at anthesis
flag leaf blade at milk grain stage
flag leaf blade (senescence) at Dough
flag leaf blade (senescence) at Ripening
leaf ligule at Full boot
Internode sec at Ear emergence
Internode sec at 30% spike
Internode sec at milk grain stage
peduncle at Ear emergence
peduncle at 30% spike
peduncle at milk grain stage
spike at Full boot
spike at 30% spike
spikelets at 30% spike
lemma at Ear emergence
lemma at milk grain stage
awns at Ear emergence
awns at milk grain stage
glumes at Ear emergence
glumes at milk grain stage
anther at anthesis
stigma & ovary at anthesis
embryo proper at Dough
endosperm at Dough
grain at milk grain stage
grain at Soft dough
grain at Hard dough
grain at Ripening
